# Optimizing UV-A Solar-Powered Lights to Enhance Lures for Codling Moth, *Cydia pomonella* L. (Lepidoptera: Tortricidae)

**DOI:** 10.3390/insects17040354

**Published:** 2026-03-24

**Authors:** Alan Lee Knight, Esteban Basoalto

**Affiliations:** 1 Instar Biologicals, Yakima, WA 98908, USA; 2 Facultad de Ciencias Agrarias y Alimentarias, Instituto de Producción y Sanidad Vegetal, Universidad Austral de Chile, Valdivia 5090000, Chile

**Keywords:** apple, pear ester, sex pheromone, pear, monitoring, natural enemies, UV LED

## Abstract

Field trials were conducted to define several parameters associated with adding LEDs to monitoring traps for codling moth (CM), *Cydia pomonella* (L.), using both a sex pheromone lure (PH1X) and a non-pheromone lure (CM4K). Traps with LEDs emitting at a peak of 395 nm with 1000–2000 mW/m^2^ were the most effective. Lights with greater intensities caught similar numbers of CMs and significantly more non-targets. Adding the UV-A lights did not increase moth catches early in the season with either the PH1X or CM4K lures. However, UV-A LEDs, when used with these two lures, significantly increased total moth catches 7- and 3-fold in July and August, respectively. The addition of the UV-A LEDs allowed CM4K-baited traps to perform significantly better in previously limiting situations, such as in weedy orchards, and in pear relative to apple. Distance from the light source is a key factor affecting light energy. Irradiance dropped >90% at 15 cm, which is the distance from the lure to the entrance of a standard delta trap. A smaller trap (7.5 cm radius) had a 4-fold greater irradiance at its entrance and caught greater numbers of non-targets but not CMs than delta traps without LEDs.

## Simple Summary

The integration of UV-A LEDs into traditional pheromone and non-pheromone baited traps addresses the challenge of effective monitoring for the codling moth (*Cydia pomonella*), a primary pest in fruit orchards. Monitoring adult moths in orchards treated with sex pheromone-based mating disruption technologies can be difficult. This study aimed to optimize trap efficacy by defining the ideal light wavelength and intensity while minimizing the accidental capture of beneficial natural enemies. Results indicated that LEDs peaking at 395 nm with an intensity of 1000–2000 mW/m^2^ were the most effective. Adjusting these parameters lower or higher did not increase moth capture but significantly increased the unintended catch of non-target insects. While the lights did not impact spring catches, they increased summer moth capture by 3- to 7-fold, particularly when evening temperatures exceeded the lower threshold for moth activity, 16.7 °C. Adding the light can override the impact of plant volatiles limiting moth catches, such as in weedy apple and pear orchards. These new monitoring traps can provide growers with pest data enabling more targeted and efficient interventions, such as pesticide applications. Limiting the non-target catches helps to protect the biodiversity of orchard ecosystems.

## 1. Introduction

Combination lures consisting of acetic acid, plant and microbial volatiles, and sex pheromones have been developed for key tortricid pests of tree fruit and grapes [1]. These lures catch both sexes and outperform standard sex pheromone lures under sex pheromone-based mating disruption (MD). Adding a single UV-A LED light to traps baited with these lures significantly increased the catch of both sexes [2,3,4]. The additive effect of the LED is dependent on the relative attractiveness of the volatile lure. For example, when used with a moderately effective lure, such as 2-phenylethanol and acetic acid for the eye-spotted budmoth, *Spilonota ocellana* (Denis and Schiffermüller), total catch was increased 11-fold [2]. Adding the UV-A LED to traps with the 7-component lure targeting the oriental fruit moth (OFM), *Grapholita molesta* (Busck), increased the secondary catch of codling moth (CM), *Cydia pomonella* (L.), up to 12-fold [3]. These two examples are the exceptions and typically, the LED addition increased moth catches 1.5–3.0-fold compared with an effective volatile lure used alone [2,3,4].

The overall effectiveness of a host plant plus microbial volatile-based lure can be variably impacted by the volatile profile within the crop [5]. A four-component non-pheromone lure (PHEROCON^®^ MEGALURE CM DUAL 4K, Trécé Inc., Adair, OK, USA) named ‘CM4K’ composed of acetic acid and three plant volatiles can outperform sex pheromone lures for CM monitoring when used under MD [6]. However, two conditions were identified that limited its relative effectiveness to a sex pheromone lure when used in pear orchards and in weedy apple or pear orchards [7]. Previously, pear ester lures were thought not to be as effective in pear compared with their use in apple or walnut orchards due to olfactory competition from key background pear volatiles [8]. However, later studies in pear showed that pear ester was effective for CM except in late season ripening ‘Bartlett’ pear with moderate to high levels (5.0–20.0%) of fruit injury [9]. Levels of fruit injury in the pear orchards monitored with the CM4K lure were not reported in a more recent study [7]. The second situation affecting the relative performance of the CM4K lure was in irrigated orchards without typical weed management practices, i.e., regular mowing, herbicides, and physical rouging. The likely elevated atmospheric concentrations of generic plant volatiles, such as six carbon acetates, alcohols, and aldehydes; and linalool and hexyl butanoate were found to reduce CM catch when formulated as lures inside traps [10]. The effectiveness of the LED to enhance moth catches is thought to be due to pulling moths inside the lit trap that would otherwise land on the outside or in nearby foliage and not be counted [2,11]. Whether the addition of the UV-A LED could override this interference of short-range orientation of CM adults into traps by host and weed volatiles has not been tested.

Several other factors can influence the effectiveness of using an LED to improve CM monitoring. First, the light is only effective when the background level of light is low. CM adults are most active for a few hours starting at dusk [12,13]. Adult activity has a minimum temperature threshold for sustained flight and sexual behaviors [14]. The minimal thresholds for light and temperature restrict the time period when a light placed in a trap could be effective. For example, early in the season (late April to mid-June) we anticipate that the LED may be less effective for CM monitoring. Secondly, several studies have demonstrated that UV-A wavelengths (395–405 nm) are more effective than blue (450 nm) or green (540 nm) for several tortricid species; however, lights with shorter UV-A wavelengths have not been studied with tortricids [2,3,4].

Light intensity is another factor that has been shown to be important in attracting insects into traps [15]. Intensity can be modified by increasing the power of the light by using more than one LED. Traps with more powerful lights are used to survey flying insects both spatially and temporally and require larger batteries than our low-cost adapted ‘yard’ and ‘mosquito zapper’ lights [16,17,18,19]. However, light intensity can also be increased by shortening the distance from the light-lure placement to the opening of the trap where the moth first enters. This distance has been 15 cm with the large delta traps used in previous studies, and smaller traps have not been tested with CM.

Previous studies with LEDs used for tortricid monitoring have reported variable results, for the unintended catch of non-targets. Catching non-target insects in orange delta traps in orchards is generally not a problem, and they were partially adopted to reduce the catch of pollinators [20]. Alternatively, non-target catch can sometimes be larger than the target species, as in the case of the European grapevine moth, *Lobesia botrana* (Denis and Schiffermüller), using white delta traps [4]. LEDs have attracted sizeable numbers of aquatic insects such as chironomids and caddisflies from nearby ponds, rivers, and canals, and filth flies from adjacent dairies and farms with livestock in some studies [2,21]. Increasing the intensity of the light could have a significant impact on the catches of non-targets [15]. Thus, a balance in attracting targets versus non-targets needs to be reached with the proper light selection [22].

Among the non-targets caught in traps placed in orchards can be several important natural enemies (coccinellids, mirids, and neuropterans) for key secondary pests of tree fruit pests, such as aphids, psyllids, mites, mealybugs, thrips, and leafhoppers [23,24]. Traps baited with host plant volatiles and acetic acid have been used to monitor natural enemies in tree fruits and other adjacent crops such as hops [25,26]. Color traps with or without LEDs have been used to attract natural enemies in greenhouses and crops [27,28,29]. However, monitoring natural enemies in tree fruits with the combination of volatile lures and LED light has not been reported.

Growers who have adopted the use of CM MD have largely discontinued using a standard 1.0 mg sex pheromone red rubber septa and have switched to higher load lures to allow better tracking of moth flights [30]. Growers’ expectations in using the 1.0 mg lure have been that CM MD should shutdown male catch in these baited traps [31]. The combination of sex pheromone and light has been widely reported with moths in other crops [32,33]. With CM, the UV-A LED was used with a combination pear ester-sex pheromone lure and increased catch 2-fold [2]. Adding an LED to traps with a standard sex pheromone lure has not been reported for CM or other tortricid pests.

Herein, we report results from studies addressing each of these important factors. First, the effect of adding a UV-A LED on male catch with either the 1.0 mg sex pheromone or total and female catches with the CM4K dual sex lure were evaluated early and late in the season during 2025. Second, the catch of male and female CMs with eight different LED lights with variable irradiances combined with the CM4K lure were evaluated seasonally. This included characterizing the catch of key natural enemies and other non-targets in four trials in organic and conventional apple and pear. Third, studies were conducted in apple and pear orchards with and without standard weed management in both 2024 (no LED) and 2025 (with LED).

## 2. Materials and Methods

### 2.1. LEDs

Eight types of lights were included in the study (Table 1; Figure S1). Solar-powered ‘yard lights’ or ‘mosquito zappers’ were purchased online through Alibaba from five manufacturers in China (Luoyang Yaoling Business Centre (Luoyang, China); Guangzhou Jiguang Lighting Co. (Guangzhou, China); Shenzhen Update Electronics Co. (Shenzhen, China); Oslon Technology Co. (Shenzhen, China); and Ningbo Chiju Import and Export Co., (Ningbo, China)). The BGR variable light was purchased from Shenzhen Lenora Lighting Co. (Guangdong, China). The green LED was purchased through Amazon (Alepod Store). The white LEDs in yard lights were replaced with purple LEDs, peak wavelengths at 395 or 365 nm (Shenzhen Chanzon Technology (Shenzhen, China) and Ningbo Jiatong Optoelectronic Technology Co. (Ningbo, China), respectively). The electrocuting wire was removed from the zapper lights. All lights had new batteries inserted at the beginning of the season. Lights were hot glued to orange delta traps so that the LED was positioned inside one of the side panels (Figure S1). The UV10 used a 2 × 5 array (3.0 cm × 2.3 cm) of SMD LEDs mounted directly on a printed circuit board placed inside the dome of the trap.

The eight LEDs in our study were chosen to allow testing of different factors affecting moth attraction, i.e., peak wavelength and irradiance. Units came with variable-sized solar panels and battery capacities (Table 1 and Figure S1). Six UV lights had peak wavelengths between 365 and 397 nm. The UV1b, UV1c, and UV1d all had a single LED, which was switched out from white to purple for our studies. The UV1a had two lights (purple and white) and only the purple UV LED was switched on during the studies. The UV2 had three LEDs (2 purple and 1 white) and only the two purple UV LEDs were used. The UV10 had 10 lights of an SMD type of LED, and the battery was inaccessible. The BGR unit had a single LED that switched between red (627 nm), green (515 nm), and blue (466 nm) every 5 s. The green LED had a single LED with a peak wavelength of 514 nm. The peak irradiance of the UV365 LED was extrapolated with data collected from ≥380 nm.

The spectral characteristics of the LEDs were measured using a Licor Spectrometer Model LI-180 (LI-COR Biosciences, Omaha, NE, USA). Measurements were conducted with lights placed inside a black cardboard box measuring 27 cm × 19 cm × 13.5 cm (L × W × H). The optical sensor was positioned inside the box with the light and connected to the outside via a Type-C USB cable for remote measurement. The spectrum intensity (mW/m^2^) was measured for each LED at two distances, adjacent to the LED (<1-cm) for all lights and at the distance from the center of the trap’s liner to the entrance of the delta trap (15 cm) or the round trap (7.5 cm).

### 2.2. Lures and Traps

Two proprietary lures were used in these studies, a binary lure to sample male and female CMs (PHEROCON^®^ MEGALURE CM DUAL 4K) referred to as ‘CM4K’, and a sex pheromone red septa lure loaded with 1.0 mg codlemone, (*E*,*E*)-8.10-decadienoate, referred to as ‘PH1X (Trécé Inc., Adair, OK, USA). CM4K consisted of a black PVC matrix loaded with pear ester, (*E*,*Z*)-2,4-ethyl decadienoate, (*E*)-4,8-dimethyl-1,3,7-nonatriene, and pyranoid linalool oxide (6-ethenyl-2,2,6-trimethyloxan-3-ol) used in combination with a white membrane dispenser loaded with acetic acid. Orange delta traps (27 cm × 20 cm) (PHEROCON^®^ DELTA VI, Trécé Inc.) with a hot-melt sticky liner (CleanBrake^®^, Trécé Inc.) were used without lights and with all the LEDs, except with the UV10 trap. The delta trap had 42 cm^2^ openings at each end. The light inside the UV10 trap was mounted in the center of a round trap with a 19 cm diameter and had three 50 cm^2^ openings separated by 5 cm solid panels (Figure S1). This commercial trap is marketed for fleas and mosquitoes and had a similar sticky liner to those used in the delta traps.

All traps were attached to poles and placed in the canopy at ca. 3 m. Orchard row was considered a replicate, and one replicate of each trap type was randomly spaced 30 m apart beginning 20–25 m from the orchard’s border. Replicates ranged from 5 and 13 among Trials 1 to 8, which compared LEDs; Trials 9 and 10, which looked at weedy and non-weedy orchards, had 27 to 53 replicates (Table 2). Traps were spaced to maximize the diagonal distance of traps between rows. Lures were placed in the center of liners. CMs on liners were counted and sexed in the laboratory. Non-targets ≥ 2-mm were counted and classified on liners in Trials #4–7.

### 2.3. Orchards

Ten trials were conducted in apple and pear orchards during 2024 and 2025 (Table 2). Orchards were either farmed conventionally or according to organic regulations. Study sites were situated near Tieton (46.7045° N, 120.7554° W) and Wapato (46.4476° N, 120.4203° W), WA (USA). The two growing areas have different crop and pest phenologies due to differences in elevation, with Tieton being colder (586 m above sea level) than Wapato (260 m above sea level). Orchards had mature canopies 3.0 to 4.0 m in height, and planting densities varied from standard central leader architecture at 450–750 trees ha^−1^ to high-density trellis systems with up to 5000 trees ha^−1^. Orchards included the most common apple and pear cultivars planted in central Washington. These included Gala, Fuji, Honeycrisp, Ambrosia, Golden Delicious, Red Delicious, Cosmic Crisp, Cripps Pink, Jazz, and Envy apples; and Bartlett, D’Anjou, Comice, and Bosc pears.

Orchards were all treated with one of two MD dispensers for CM. Both dispensers were loaded with codlemone and pear ester: CIDETRAK^®^ CMDA COMBO MESO loaded with 850 mg codlemone and 500 mg pear ester and applied at 80 dispensers ha^−1^, and CIDETRAK^®^ CMDA COMBO PP with 90 and 60 mg of codlemone and pear ester applied at 1000 dispensers ha^−1^, respectively (Trécé Inc.). Placement of the high-density hand-applied dispensers typically began <5 m from the edge of the block and were applied on every row at a spacing of 2 to 3 m depending on tree density, canopy closure, and row spacing or at 11 m spacing with the MESO dispenser. All orchards were sprayed by the grower with standard management and nutritional programs. Sprays applied for CM varied from 1 to 5 applications of diamide, spinosyn, and neonicotinoid chemistries in conventional orchards. Organic orchards were treated with multiple applications of granulosis virus, horticultural oil, and 0–4 sprays of a spinosyn insecticide.

Dusk times and hourly air temperatures were accessed from two proprietary weather stations situated near the clusters of orchards in Tieton and Wapato, from 1 May to 1 September 2025. Data were summarized monthly for May through August. Dusk occurred at 20:11 h on 1 May and 19:38 h on 1 September, creating 9.37 and 10.46 h of darkness between sunset and sunrise, respectively.

### 2.4. Trials

#### 2.4.1. Early Versus Late Season Use of LEDs

Two trials were conducted in organic apple orchards near Wapato to compare male CM catch in delta traps with the PH1X lure alone and in combination with the UV1 LED (Table 2). Trial #1 started on 20 May 2025 and ran for 13 days with 10 replicates, while Trial #2 started on 14 July 2025 and ran for 11 days with 10 replicates; two similar trials were conducted with the CM4K lure in traps early and late in the season, respectively. Trial #3 started 17 May and ran 16 days with 13 replicates. Trial #4 started on 15 July and ran for 15 days with seven replicates (Table 3).

#### 2.4.2. Mid- to Late-Season Use of LEDs with the CM4K Lure

Four trials were conducted with 3 to 6 LED types plus traps with no lights added using CM4K lures (Table 2). Trials #5 and #6 were conducted from mid-June to the start of July. Trials #7 and #8 were conducted later in the season from mid-July to mid-August. Trials included 5 to 13 replicates and lasted six to 34 days.

#### 2.4.3. Non-Target Catches

All non-targets caught in traps that were ≥2 mm were counted in Trials #4–7, also non-targets ≤ 2.0 mm were counted in Trials #5–8 (Table 4 and Table 5).

#### 2.4.4. Comparison of PH1X and CM4K Lures with and Without LEDs in Weedy or Weed-Managed Apples and Pears

Monitoring studies were conducted in apple and pear orchards in both 2024 and 2025 to compare CM catches in delta traps with either the PH1X or CM4K lures. Studies in 2024 compared the two lures in traps without LEDs (Trial #9), while studies in 2025 compared the two lures with UV1 LEDs (Trial #10). The monitoring protocol was the same as described for studies conducted from 2021 to 2024 [34,35]. Replicates were pairs of traps placed randomly 30 m apart down orchard rows. Replicates were established as one pair of traps per 2 ha. Replicates were characterized as organic or conventional, apples or pears, and either with minimal weed management (organic) or seasonal applications of herbicides (conventional) or physical rouging of orchard rows (organic) (Figure S2). Gas-powered string ‘weed eaters’ were used in the weedy organic orchards under trees to remove weeds surrounding the under-tree irrigation systems. Weeding was conducted in these orchards in late June and again in mid-August. Drive roads in all orchards were mowed two times in weedy orchards and three times in weed-managed orchards during the season. Moth catches were summarized in 2024 across both crops and both weed management types from 1 May to June 30 and from 1 July to 31 August. The 2025 study monitored the non-weedy orchards over the same two time periods. Weedy apple and pear orchards in 2025 were monitored from 1 July to 31 August.

### 2.5. Statistical Analysis

Statistical analyses of the catches in traps were performed with R software v. 4.4.2 [36]. Moth catch data were expressed as catch per day in each experiment. The experimental design was randomized with orchard rows considered as replicates. Data were analyzed according to their distribution. Data normality was tested with Shapiro–Wilk’s test and Levene’s Test for Homogeneity of Variance. A linear model (*lm*) was used with normal data, while data found to be close to a normal distribution were transformed using the square root (*sqrt(x)*). Data that were not normally distributed were found to fit a Poisson distribution, and a generalized linear model (*glm*) was used. Akaike’s information criteria (*AIC*) and residuals were both used to select the fitted models in each analysis. A multiple comparison post hoc test was performed on the fitted models (*glht* function from *multcomp* package) and Tukey’s HSD test (*p* < 0.05) was used to discriminate significant differences among treatments. Outputs reporting *F*-statistic refer to normal data, while outputs reporting the X^2^ statistic refer to data not normally distributed. Data are reported as mean values ± standard error (SE). Moth catches in paired delta traps baited with either the PH1X or CM4K lures in both years were analyzed with a paired-sample *t*-test. Treatments with zero catches were excluded from the analysis. Hourly temperatures for 2025 collected from proprietary sites situated near each orchard cluster in Tieton and Wapato were analyzed with descriptive statistics.

## 3. Results

### 3.1. LEDs Performance

The irradiance of the eight lights measured at 0.5 cm from the source varied among units (Table 1). The irradiance of the six units with UV-A LEDs varied 3-fold. The UV2 unit with two LEDs had the highest UV irradiance. The UV10 unit, despite having 10 SMD LEDs, had the lowest irradiance when measured at the center of the 2 × 5 grid. UV1d had the smallest battery and had a similar low irradiance. The intensity of the BGR LED was similar among the three colors. The most intense light was the green LED, approximately twice as bright as the UV2 but with a different spectrum.

The percent reduction in the irradiance of all the LEDs placed in the orange delta trap dropped 98.5–99.8 when measured at 15 cm from the source. This strong reduction would correspond to an irradiance of 17 to 35 mW/m^2^ among the five UV1 and UV2 LEDs at the perimeter of the delta trap. The drop-off of the red, green and blue lights irradiance was even stronger, with only 2–13 mW/m^2^ measured at the same distance. Due to the smaller radius (7.5 cm) of the round trap, the irradiance from the UV10 LED was 125 mW/m^2^ at the distance to the perimeter of the trap or nearly four times brighter than the other LEDs at the opening of the delta trap (Table 1).

### 3.2. Post-Dusk Hours Warmer than Threshold for CM Flight Activity

Hourly temperature data recorded from the two weather stations after dusk from 1 May to 31 August were consistent with their elevational differences. The accumulated hours after dusk and before sunrise that were above the activity threshold for CM was 434 h in Wapato (lower elevation) and 311 in Tieton (higher elevation). CM flight after dusk was most limited by temperature in May, with 17 and 15 days restricting flight completely (maximum daily temperature < 16.7 °C) and 10 and 14 days with partial restrictions (<4 h above threshold after dusk) of flight in Tieton and Wapato, respectively. Evening temperatures in June warmed and only 2 and 1 days were too cold for any CM flight after dusk and 12 and 16 days had some temperature-based restrictions imposed on the duration of CM flight for Tieton and Wapato, respectively. In comparison, over the 62-day period during July and August, only 5 and 8 days had some temperature restrictions (<4 h) on CM flight after dusk.

### 3.3. Early Versus Late Seasonal Use of LEDs

The effectiveness of the UV-A LED added to traps baited with the 1X PH lure was different depending on the seasonal timing (Table 3). The addition of the light did not influence male catches in traps with the PH1X lure in May–June. In comparison, CM male catches were 7-fold higher in traps with the UV LED in July. Trials #3 and #4 evaluated the use of the UV1 LEDs early and late in the season in traps with the CM4K lure. The addition of the LED did not enhance total or female moth catches in the early trial (Table 3). In comparison, the late-season trial had significantly more (4-fold) total moths caught with the LED and the traps without LEDs failed to catch any females.

### 3.4. Mid- to Late Seasonal Use of LEDs

Studies were conducted from mid-June to July to compare traps with or without LEDs added to the use of the CM4K lure. The most effective trap type in Trials #5 and #6 caught 3- to 4-fold as many total and female moths as traps without LEDs (Table 4). The UV2, UV1a, and UV1b in Trial #5 caught more total moths than traps with no LED. The UV2 and UV1a LEDs were the only two that caught significantly more female CM than traps with no LEDs. The UV2 LED also caught significantly more total CMs than traps with a green LED or the BGR LED. The BGR, UV10, and the green LED traps did not catch more CMs than traps with no lights. All three UV LEDs in Trial #6 caught significantly more total and female moths than traps with no LED and did not differ among themselves.

Two additional trials to compare LEDs with the CM4K lure were conducted later in the season between mid-July and mid-August (Table 5). Trial #7 in apple compared six treatments, and the UV1b and UV2 caught more total and female and only total moths compared with the trap with no LED, respectively. No differences in catch were found among the five LED treatments. Trial #8 in pear compared five LEDs against no LED, and only the UV1d caught significantly more total and female CMs than the no LED treatment. No differences were found among the LED treatments.

### 3.5. Non-Targets Including Natural Enemies

All non-targets caught in traps that were ≥2 mm were counted in Trials #4–7. The most important natural enemies were the anthocorid bug, *Deraeocoris brevis piceatus* (Knight), a green lacewing, *Chrysopa nigricornis* Burmeister, and a brown lacewing, *Hemerobius* spp. Incidental catch of coccinelids, snakeflies, and spiders occurred. A miscellaneous group of non-targets included: unidentified muscid, chironomid, and tipulid dipterans; hemipterans including *Colladonus* spp., other leafhoppers, and the tarnished plant bug, *Lygus lineolaris* (Palisot de Beauvois); two unidentified species of trichopteran caddisflies; and various species of unidentified moths. Many moth species of similar size as CM (ca. 10 mm) were caught across studies. An occasional tortricid pest, oblique banded leafroller, *Choristoneura rosaceana* (Harris), was caught in traps. Most of the smaller moths (<6 mm) were gracillariids, including the western tentiform leafminer, *Phyllonorycter elmaella* (Doganlar and Mutuura). Larger moths (>14 mm) were primarily noctuids.

Non-targets ≤ 2.0 mm were counted in Trials #5–8 (Table 4 and Table 5). At least one type of LED-baited trap caught significantly more total non-targets than delta traps with no LEDs in each trial. However, the density of non-targets versus CM total catch varied 60-fold among trials. Traps placed in organic pear orchards in mid-June caught large numbers of *D. brevis* across all seven treatments in Trial #5. *D. brevis* accounted for 91% of all catches (Figure S3). Specifically, the UV10 traps caught significantly more non-targets and *D. brevis* than delta traps with no LED. The green LED had the highest catch ratio of non-targets to CM moths.

Traps performed differently in conventional apple orchards in Trial #6 during June. The catch of *D. brevis* was 25–175-fold lower in delta traps with the three LEDs used in both trials and did not differ among these three treatments or when compared with traps with no LEDs (Table 4). Only one trap type (UV2) caught significantly more total non-targets than the trap with no LED and the other two traps with UV1 lights. The most abundant non-target in this trial was chironomid adults accounting for 51% of all non-targets.

Non-target catches in organic apple replicates were low in both Tieton and Wapato orchards from mid-July to mid-August in Trial #7 (Table 5). The addition of either the UV2 LED to a delta trap or the UV10 trap significantly increased the total non-targets compared with delta traps with no LEDs, 9- to 10-fold higher. *D. brevis* catches were consistently low across treatments at ≤0.2 per day. The most abundant non-targets on liners were leafminers and midges, accounting for 58% of the total. The BGR LED had the lowest catch ratio of non-targets and CM adults.

The final non-target study (Trial #8) was conducted in July in conventional pear orchards. *D. brevis* was the most abundant non-target, but daily catches were much lower than counts from the organic pear study in Trial #5. Both the UV2 and UV10 LEDs caught significantly more *D. brevis* and 4- to 8-fold more non-targets than traps without an LED, respectively.

### 3.6. Use of LEDs in Weedy and Cultivated Apple and Pear Organic Orchards, 2024-25

The mean total moth catches between traps baited with either the CM4K or the PH1X lures were about 2-fold higher in 2025 with the use of UV LEDs than in 2024 when traps did not have lights added (Table 6). Traps in 2024 (Trial #9a) baited with the CM4K lure in apple orchards with managed weeds caught 3–8-fold more total moths than similar traps with the PH1X across the two flight periods (Table 6). Results were similar in pear orchards (Trial #9b) with weed management, but only significantly different in the 1st flight. During both flights and in both crops with unmanaged weeds, there was no difference in mean moth catch between the two lures. Levels of pear injury from CM prior to harvest were <0.5% across the pear orchards.

The study was repeated in 2025, adding LEDs to all traps, and was only conducted during the second flight in the weedy apple and pear orchards (Table 6). Traps in apple and pear orchards with weed management in both apple and pear had significantly higher total counts (5- and 3-fold, respectively) with the CM4K versus the PH1X lure. However, unlike in 2024, apple and pear orchards with unmanaged weeds also had significantly higher counts with the CM4K than the PH1X lure during the 2nd flight (4- and 2-fold, respectively). Mean levels of pear fruit injury in the weedy orchards were lower in 2025 than 2024, <0.2%. In comparison, fruit injury from CM was higher in some pear orchards with weed management due to variable immigration, 0.0 to 5.0%.

## 4. Discussion

The use of LEDs with their associated benefits over previous more cumbersome lights has generated new methods to use insect’s visual attraction to improve pest, natural enemies, and biodiversity monitoring and management [32,33]. Incorporation of low cost, solar-powered LED ‘yard lights’ and ‘mosquito-zappers’ with several dual sex lures for tortricids has created an approach to develop more effective trapping strategies for this important pest group [2,4]. The trap prototype we have developed is inexpensive because it is adapted from commercially available lights. The lights are powered by a small solar panel and use either one AA or AAA battery and typically have adequate storage for 10–12 h of illumination per day all season. Traps with proper off-season storage and new batteries have been used for up to 3 years in tree fruits in Washington State, USA.

Lepidoptera adults exhibit three peaks in their spectral sensitivities: UV (355 nm), blue (440 nm), and green (525 nm) that can vary in their relative amplitudes [37,38]. Interestingly, a lower sensitivity to UV than green light does not necessarily correlate with behavioral impacts of light within the host crop [39]. For example, the peak behavioral response to different lights, such as with *G. molesta* and *L. botrana*, occurs at 395–405 nm despite a second broader peak of photoreceptors responding to the green spectrum [4,39,40]. Also, species within a single family can have different responses, i.e., seven tortricids were most attracted to UV at 370 nm, while three species were caught more readily in traps with a green light at 578 nm [41]. Trap catch of CMs with a dual sex lure exhibited a similar pattern with UV-A > green > blue [2]. Our current study with CM supports the greater behavioral impact of UV-A vs. green light.

Combinations of UV and green LED lights activating more than one photoreceptor have significantly improved trapping with a variety of insects. These include the true bug, *Nezara* spp., the dipteran, *Lycoriella ingenua* [42], and the psyllid, *Diaphorina citri* (Kuwayama) [43]. However, the UV plus green combination did not increase catch of the Indian meal moth, *Plodia interpunctella* (Hübner) [44]. The addition of a UV light alone or with a green LED in combination with green, blue, or yellow sticky traps have also been effective with thrips, whiteflies, and psyllids [43,45,46]. The 3-color variable light in our study did not catch more CMs but did not include a UV light. Previously, we found that wiring additional LEDs to the commercial single LED lights decreased their intensity. However, modifying the UV2 light to include both a green and UV LED could be tested.

LEDs emit a narrow band of light, usually about 60 nm wide, so that the spectra from UV-A to violet LEDs significantly overlap [47]. Several studies have compared closely spaced peak wavelengths in this spectral range for moths [48,49,50,51,52] or natural enemies [22,27,29,49]. In general, they have found that attraction is similar across this narrow spectrum (365–420 nm). CMs and non-targets responded similarly in our current study to traps with the CM4K lure and either a 365 or 395 nm LED. Traps with LEDs emitting a peak emission < 365 nm have not been tested with tortricids or in tree fruits.

Insects’ perception of the intensity of a light source is impacted both by the LED’s characteristics and its distance from the light [33]. In general, insect attraction is increased with light intensity to some level where further increases can reduce catch, i.e., almond moth, *Cadra cautella* (Walker) [53]. Traditional light traps used a high-intensity source (up to 200 W) and required heavy storage batteries or a direct power source [54]. Even with the development and adoption of LED lights due to their several advantages (energy efficiencies and an adjustable, narrow light spectrum), the intensity of lights remained relatively high to maximize the magnitude of catches [55]. Studies have used as many as 150 LEDs with 15 W to survey moths as effectively as the standard mercury-vapor and Rothamsted incandescent lights [56].

Early studies utilizing LED light traps considered 6–10 W as a ‘weak’ or ‘dim’ source and estimated they had a drawing range < 10 m [17,57]. An early development of a more portable LED-based trap measured the irradiance of its 10 W lamp at 50 cm as 1430 mW/m (a value similar to our ‘at source’ measurements), which would be a light source nearly 25-fold more intense according to the inverse-square law than at the entrance of our delta traps at 10 cm [47]. Our studies have deliberately employed only a single LED (≤1 W) to minimize the range of attraction for non-targets and only pull male and female targets into the trap who were already attracted to the area by the lure [2]. Testing the UV2 trap with two LEDs with nearly twice the intensity as the single LEDs had a minimal result on CM but often caught more non-targets.

Size and shape are key factors influencing trap performance including those adding a UV LED. Among our traps, the distance of the nearest LED in the 2 × 5 array in the UV10 trap was approximately half the distance of the single LED from the entrance of the delta-shaped traps, thus the UV10’s irradiance was 4-fold higher at its perimeter. The significantly greater non-target catches with this trap in several trials was likely in response to this greater light intensity. However, it did not catch more CM adults. The physical differences in the round and delta traps, including the shape, color, and area of entrances into the trap, were also likely important factors affecting insect catches. Evaluations of shorter delta traps would likely be a more robust comparison of intensity’s effects on both CM and non-target catches.

Several external factors are known to influence the performance of light traps including atmospheric parameters, such as moonlight and cloud cover, and daily variability in temperature and wind speed [58,59]. Important crepuscular pest species such as CM have been well studied, including their circadian periodicity, and the influence of wind speed and temperature on moth activity are well understood [12,14,60]. We found that the addition of the LED did not improve either a sex pheromone or kairomone lure when used early in the season when flight time temperatures for CM were below their activity threshold. Similarly, the LED did not prove effective with the PET+AA lure for *L. botrana* during the 1st moth flight in Chile [4]. Previous tortricid studies in North America were all conducted from the middle to the end of the summer season [2,3].

Few non-targets occurred in traps baited only with the four-component CM4K lure. There is some information on the attractiveness of the lure’s individual components for various non-targets. The attraction of pear ester for other tortricids and key moth pests in other crops has been reported, but not its possible effect on natural enemies [61,62]. The component, DMNT, was found not to be attractive to any beneficial insects in hops, *Humulus lupulus* L., including *D. brevis* [25]. Linalool is a common floral volatile with known attraction to moth and bee pollinators as well as being a key moderator of tri-trophic interactions [63]. However, few studies have evaluated the biological activity of linalool oxide. Linalool oxide was identified in the bouquet of volatiles from grape *Vitis vinifera* L., flowers and in apple [64] and has been investigated as a minor component of a blend for *L. botrana* [65]. The important inclusion of the acetic acid co-lure in our various lures for tortricids [66] could have some influence on the catch of non-targets, such as syrphids [67].

The influence of trap color on performance has been investigated for various tortricids but generally has not been found to be important with sex pheromone lures [68,69,70]. In comparison, clear plastic traps were found to be more effective for CM, grape, and peach pests using either sex pheromone-based or kairomone lures [71,72,73]. The broad adoption of orange traps for CM monitoring followed a demonstration of it being more effective than white traps when used with pear ester-based lures, especially for females active at dusk [20]. In addition, trap color can be important for protecting pollinators [68,69] and minimizing the catch of non-targets such as muscid flies [69]. The UV10 was a white-grey color but did not catch higher levels of hymenopterans or fewer dipterans than orange traps. Our previous study with *L. botrana* used white delta traps, and traps often had much higher counts of non-targets than the target pest [4].

Trap liner saturation can be a problem with some monitoring programs due to either large captures of the targeted pest or the associative catch of non-targets [21,74]. Non-saturating traps with various retention methods are a useful substitute [75]. Unfortunately, efforts to enhance the catch of tortricids using UV lights can potentially increase liner saturation issues with the target and non-target catches. The use of smaller traps could exacerbate this issue. Screens have been developed to reduce non-target catches but can also reduce target catches [76]. Screens would likely be ineffective with CM due to similar sizes of the most abundant non-targets, and the target in our study was found with *G. molesta* and muscid flies [21]. Possible improvements to the traps, such as using diffused or reflected light inside the trap, have been demonstrated for some insects but have not yet been evaluated for tortricids [77,78].

The catch of non-targets in our studies was generally low relative to the numbers of CMs (Figure S4). The key non-targets caught in our studies were all groups known to be attracted to UV light, i.e., muscid [79] and chironomid Diptera [80], Trichoptera [81], Hemiptera [15] and Lepidoptera [37,82]. Significant extra orchard sources of non-targets can occur in central Washington from riparian areas, other crops, and water bodies [83,84,85]. Orchards in the irrigated central WA region are generally planted near rivers, and there is an extensive system of canals and pond reserves on most farms. Adjacent crops in this arid region can include a diversity of orchards and irrigated field crops, such as alfalfa hay, potato, wheat, grape, hops, onion, and corn [86]. This diverse array of crops typically has their own key pests but can share more broadly a complex of generalist predators.

The generalist predator guild in central Washington includes several key species of spiders, predatory true bugs, ladybeetles, and lacewings [24,84]. Only three groups of natural enemies were caught regularly in our traps, the mirid *D. brevis* in often large numbers, the chrysopid green lacewing *C. nigricornis*, and the hemerobiid brown lacewing, *Hemerobius* spp. All three are known to be attracted to UV light [87,88]. No syrphids were caught and they are not attracted to UV light [89]. Surprisingly, few coccinellids were caught across all trials despite their known attraction to UV light [90]. The paucity of generalist predators caught in these studies may have been because the selected orchards did not have noticeable populations of their key prey: green aphids *Aphis* spp., rosy apple aphid, *Dysaphis pantaginea* Passerini, or woolly apple aphid, *Eriosoma lanigerum* (Hausman) [23].

One benefit of switching to LED-based traps from fluorescent lights was being able to target specific pests without removing excessive numbers of non-targets [91]. A proper selection of the LED spectra can help to maximize the removal ratio of pests to natural enemies [22]. However, the absolute numbers of natural enemies in this later study did not vary in traps with 395 to 616 nm LEDs. *D. brevis* was the most abundant natural enemy on traps in our studies and was caught with either UV or green LEDs.

Mirids are both pests and natural enemies in many crops. *D. brevis* is a key predator in both apple and pear [23]. Herbivorous mirids develop in various weeds and cultivated plants, and do not reproduce in orchards but are occasional pests injuring fruits [23]. Sex pheromones have been identified for several mirid species [92]. The European tarnished plant bug (ETPB), *Lygus rugulipennis* Poppius, is an important pest of a wide variety of cultivated crops in Europe [93]. The sex pheromone has been used successfully with hexyl butyrate and phenylacetaldehyde in a push–pull strategy for this pest in strawberry [94]. However, light has not yet been used as part of the ‘pull’ in this system. ETPB was not well trapped with a water trap plus a UV-A LED, but a white light was effective [95]. A combination of colored sticky cards baited with sex pheromone was effective with the related *Lygus lineolaris* [96]. Whitefly management with the commercially reared mirid predator *Nesidiocoris tenuis* in greenhouses was improved with supplemental light from 365 to 405 nm [50].

The large catches of *D. brevis* on liners were interesting but not uncommon. We have observed over the past decades that liners will occasionally have large counts of this insect, suggesting that there is some factor influencing their aggregation on the liner (A.L.K., *unpublished data*). Aggregations of *D. brevis* also occurred on liners in traps with no lights but were significantly greater in traps with LEDs. Mirids can communicate via sex pheromones and through substrate-borne vibrations [92,97]. Neither modality has been studied with *D. brevis*. The mirid *Campylomma verbasci* (Meyer) is another common and important predator in these orchards, and a commercial sex pheromone blend has been developed, but it did not show up in our traps [98]. Unfortunately, we did not sex *D. brevis* caught on the liners. Female tortricids including CMs freshly caught on liners can release pheromone and attract conspecific males [99,100]. This response has not been documented for other orders of insects caught in traps with sticky liners.

While total and female moth catch can be increased ca. 3-fold with the use of a single UV LED with several tortricids, pest managers do not necessarily want to process more moths. However, they do want to increase the precision and predictive value of traps for levels of oviposition and potential fruit injury. Increasing the catch of female moths allows researchers to evaluate mating success under MD and consider the role of immigration on integrated pest management programs [34,35]. Studies conducted in Chile and Spain with EGVMs have found a range in the female population mating status in different vineyards and at different points in the season [4].

The ability to catch more female tortricids offers some promise to develop effective MD combined with female removal programs [101,102]. A male-bias sexual difference in moths’ responses to light traps has been noted in several studies [103,104,105]. However, no similar pattern has been found using UV LEDs with dual sex lures with tortricids [2,4]. Our current results with CMs were similar. Also, the addition of the UV LEDs in these studies has not impacted the proportion of unmated females caught with dual sex lures.

These studies used traps at 60 ha^−1^ and attained a moderate level of injury reduction (<60%). In comparison, a recent study in Turkey using only UV lights (350–400 nm, 20 W) at 20 traps ha^−1^ did not reduce fruit injury [106]. Unfortunately, operational factors including the cost of commercial traps and lures is still quite high when used at densities of 60–120 traps ha^−1^. Future loss of insecticide efficacy for both conventional and organic growers for CM would likely make this approach more attractive [107,108].

Adopting the low-cost UV LEDs could potentially be used to enhance other aspects of pest management in tree fruit. Limb tapping or sweep netting are often used in sampling key pests and natural enemies but are subject to large variations, especially when used within tree canopies [109,110]. An alternative or supplementary sampling tool is the use of yellow or blue sticky cards [111,112]. Combinations of sticky cards with LED lights including UV or green have been developed for some sampling applications [46,95]. Two possible uses of LEDs in tree fruit could be to enhance sampling for pear psylla, *Cacopsylla pyri* [110] or for the leafhopper *Colladonus montanus reductus*, which is a vector of X-disease in stone fruit [113]. The use of multiple sensory cues for the Asian citrus psyllid *Diaphorina citri* Kuwayama including visual (color, UV reflectant), olfactory (scent lure), and gustatory (phagostimulant) lures have been combined effectively, and a similar approach should be considered for select pests of deciduous tree fruits [114,115].

## 5. Conclusions

The initial studies conducted to develop a more effective monitoring trap and lure for CMs were very close to the optimal design. The use of a single 1 W UV-A (peak at 395 nm) LED with the CM 4K lure catches the maximum numbers of male and female CMs and the minimal number of non-target organisms. Using a different peak wavelength or intensity did not increase the CM catch, but more intense lights caught more non-targets. The beneficial effect of adding the LED to traps only occurs when dusk temperatures exceed the threshold for adult flight activity.

## Figures and Tables

**Table 1:** Parameters of the eight LED lights evaluated in trapping studies of codling moth using the CM4K lure, 2025.

Light	No. LEDs Light Type ^a^	LED Source	Battery	Solar Panel (cm), Trap Shape	Peak Radiation (nm)	Irradiance (mW m^2^)	
						At LED	% Reduction at Trap’s Entrance
UV1a	1 White/1 purple	original	600 mAh, AA	4 × 4, delta	394	1600	98.5
UV1b	1 purple	Chanzon	1100 mAh, AAA	4 × 4, delta	395	2389	98.7
UV1c	1 purple	Jiatong	600 mAh, AA	4 × 4, delta	365	2300	99.1
UV1d	1 purple	Chanzon	200 mAh, AAA	3 × 3, delta	397	1179	98.6
UV2	1 White/2 purple	original	600 mAh, AA	5 × 5, delta	394	3500	99.0
UV10	10 purple	original	1200 mAh	7 × 5, round	395	1182	89.4 ^c^
BGR ^b^	1 blue/green/red	original	600 mAh, AAA	3.5 × 3.5, delta	627, 515, 466	1885, 1711, 1203	99.8
Green	1 Green	original	500 mAh, AA	6 × 6, delta	514	6636	99.8

^a^ Only the UV LEDs were switched on in the six lamps. ^b^ The BGR LED emits a repeating 5 s sequence of red, green, and blue light. ^c^ The 2 × 5 LED grid in this round trap is 7.5 cm from the entrances so the irradiance would be 4-fold greater (inverse square law) relative to 0.5 cm from the LED compared with the LEDs used in delta traps.

**Table 2:** Apple and pear orchards included in the 2025 trials with various UV LEDs.

Trial #	Year	Lures	# LEDs Types Tested	Replicates	Dates	Location	Crop	Management
1	2025	PH1X	1	10	20 May–2 June	Wapato	Apple	Organic
2	2025	PH1X	1	10	14–25 July	Tieton	Apple	Organic
3	2025	CM4K	5	13	17 May–2 June	Wapato	Apple	Organic
4	2025	CM4K	6	7	15–30 July	Wapato	Apple	Organic
5	2025	CM4K	6	8	14–20 June	Tieton	Pear	Organic
6	2025	CM4K	3	10	15 June–2 July	Wapato	Apple	Conventional
7	2025	CM4K	5	8	22 July–15 August 12 July–15 August	Tieton Wapato	Apple	Organic
8	2025	CM4K	5	5	15–25 July	Wapato	Pear	Conventional
9a	2024	PH1X, CM4K	0	50	1 May–31 August	Tieton Wapato	Apple	Organic and conventional
9b	2024	PH1X, CM4K	0	27	1 May–31 August	Tieton Wapato	Pear	Organic and conventional
10a	2025	PH1X, CM4K	1	53	1 May–30 June 1 July–31 August	Tieton Wapato	Apple	Organic and conventional
10b	2025	PH1X, CM4K	1	32	1 May–30 June 1 July–31 August	Tieton Wapato	Pear	Organic and conventional

**Table 3:** Comparison of codling moth catches in delta traps baited with the sex pheromone lure (PH1X) in Trials #1 and #2 and with the binary lure (CM4K) in Trials #3 and #4, with or without the addition of a UV LED, conducted in apple orchards in early and late season 2025.

Early Season				Late Season			
Trial #	Lure	Mean ± SE Catch per Day		Trial #	Lure	Mean ± SE Catch per Day	
		Total	Females			Total	Females
1	PH1X	0.41 ± 0.14 A	-	2	PH1X	0.23 ± 0.10 B	-
	PH1X + UV1a	0.59 ± 0.21 A	-		PH1X + UV1a	1.61 ± 0.56 A	-
Statistics		*X*^2^ = 0.34, *p* = 0.558	-	Statistics		*X*^2^ = 11.71, *p* = 0.001	-
3	CM4K	0.23 ± 0.04 A	0.13 ± 0.03 A	4	CM4K	0.13 ± 0.04 B	0.00 ± 0.00
	CM4K + UV1b	0.35 ± 0.07 A	0.19 ± 0.04 A		CM4K + UV1c	0.52 ± 0.18 A	0.21 ± 0.10
Statistics		*F*_1,24_ = 0.52, *p* = 0.478	*F*_1,24_ = 0.41 *p* = 0.526	Statistics		*F*_1,12_ = 7.92, *p* = 0.016	-

Mean values within each trial followed by different uppercase letters were significantly different, Tukey’s Test, *p* < 0.05. All trials were conducted in organic apple orchards.

**Table 4:** Comparative catches of codling moth (CM), the hemipteran natural enemy *Deraeocoris brevis*, and all non-targets (NT), across two field trials conducted in apple and pear at mid-season, 2025.

Trial #		Mid-Season				
	Added Light	Mean ± Catch per Day				Ratio NT/CM
		CM Total	CM Females	*D. brevis*	All Non-Targets	
5	None	0.48 ± 0.09 C	0.25 ± 0.06 B	3.44 ± 2.69 B	3.50 ± 2.67 C	7.29
	UV1a	1.33 ± 0.21 AB	0.73 ± 0.13 A	7.63 ± 2.26 AB	7.83 ± 2.25 ABC	5.89
	UV1b	1.38 ± 0.20 AB	0.64 ± 0.11 AB	5.13 ± 2.01 AB	5.50 ± 1.97 BC	3.99
	UV2	1.58 ± 0.27 A	0.84 ± 0.15 A	11.88 ± 1.61 A	12.67 ± 1.69 AB	8.02
	BGR	0.71 ± 0.11 BC	0.36 ± 0.10 AB	4.94 ± 1.62 AB	5.06 ± 1.61 BC	7.13
	UV10	0.98 ± 0.14 ABC	0.63 ± 0.09 AB	12.88 ± 0.94 A	14.81 ± 0.94 A	15.11
	Green	0.48 ± 0.11 C	0.27 ± 0.09 B	7.94 ± 1.52 AB	9.60 ± 1.44 ABC	20.00
Statistics		*F*_6,49_ = 7.86, *p* < 0.0001	*F*_6,49_ = 5.52, *p* = 0.0002	X^2^ = 21.652, *p* = 0.001	X^2^ = 29.890, *p* < 0.001	-
6	None	0.21 ± 0.05 B	0.09 ± 0.03 B	0.06 ± 0.06 A	0.10 ± 0.05 B	0.48
	UV1a	0.45 ± 0.06 A	0.28 ± 0.05 A	0.04 ± 0.04 A	0.16 ± 0.05 B	0.36
	UV1b	0.64 ± 0.09 A	0.34 ± 0.06 A	0.39 ± 0.39 A	0.11 ± 0.05 B	0.17
	UV2	0.50 ± 0.06 A	0.23 ± 0.03 A	0.46 ± 0.16 A	2.69 ± 0.62 A	5.38
Statistics		*F*_3,36_ = 8.21, *p* = 0.0003	*F*_3,36_ = 7.66, *p* = 0.0004	X^2^ = 3.180, *p* = 0.365	X^2^ = 49.661, *p* < 0.001	-

Mean values within each trial followed by different uppercase letters were significantly different, Tukey’s Test, *p* < 0.05. Trial 5 was conducted in an organic pear orchard, and trial 6 was conducted in a conventional apple orchard.

**Table 5:** Comparative catches of codling moth (CM), the hemipteran natural enemy *Deraeocoris brevis*, and all non-targets (NTs), across two field trials conducted in apple and pear in late season, 2025.

Trial #,		Late-Season				
	Added Light	Mean ± Catch per day				Ratio NT/CM
		CM Total	CM Females	*D. brevis*	All Non-Targets	
7	None	0.19 ± 0.02 B	0.10 ± 0.03 B	0.01 ± 0.01 A	0.07 ± 0.03 C	0.37
	UV1a	0.48 ± 0.04 AB	0.32 ± 0.03 A	0.06 ± 0.05 A	0.15 ± 0.05 ABC	0.65
	UV1b	0.65 ± 0.17 A	0.41 ± 0.13 A	0.06 ± 0.05 A	0.19 ± 0.06 ABC	0.29
	UV2	0.63 ± 0.07 A	0.31 ± 0.05 AB	0.20 ± 0.14 A	0.64 ± 0.22 AB	1.02
	BGR	0.41 ± 0.10 AB	0.22 ± 0.07 AB	0.02 ± 0.01 A	0.13 ± 0.03 BC	0.32
	UV10	0.49 ± 0.10 AB	0.31 ± 0.08 AB	0.08 ± 0.03 A	0.68 ± 0.19 A	1.39
Statistics		*F*_5,42_ = 4.24, *p* = 0.003	*F*_5,42_ = 3.49, *p* = 0.010	*X*^2^ = 6.16, *p* = 0.291	*X*^2^ = 23.54, *p* < 0.001	-
8	None	0.28 ± 0.19 B	0.12 ± 0.08 B	0.02 ± 0.02 C	0.21 ± 0.15 C	0.75
	UV1c	0.73 ± 0.26 AB	0.31 ± 0.11 AB	0.25 ± 0.24 BC	0.40 ± 0.23 BC	0.55
	UV1d	1.16 ± 0.24 A	0.63 ± 0.15 A	0.33 ± 0.26 ABC	0.46 ± 0.26 BC	0.40
	UV2	0.85 ± 0.13 AB	0.37 ± 0.07 AB	0.74 ± 0.30 A	0.84 ± 0.31 AB	0.99
	BGR	0.56 ± 0.13 AB	0.22 ± 0.06 AB	0.14 ± 0.05 ABC	0.25 ± 0.08 BC	0.45
	UV10	0.39 ± 0.11 AB	0.21 ± 0.08 AB	0.66 ± 0.27 AB	1.74 ± 0.76 A	4.46
Statistics		*F*_5,24_ = 2.98, *p* = 0.031	*F*_5,24_ = 3.73, *p* = 0.012	*F*_5,24_ = 2.92, *p* = 0.034	*F*_5,24_ = 12.20, *p* = 0.032	-

Mean values within each trial followed by different uppercase letters were significantly different, Tukey’s Test, *p* < 0.05. Trial 7 was conducted in an organic apple orchard, and trial 8 was conducted in a conventional pear orchard.

**Table 6:** Comparative catch of CM adults in delta traps baited with a PH1X sex pheromone lure or with the dual sex, CM4K lure in trials conducted in apple and pear orchards with or without effective weed management during 2024 with no LED lights (Trial 9) and in 2025 with LED lights added (Trial 10).

				May–June			July–August		
Trial #	Crop	Weedy	# Replicates	Mean ± SE Moths per Trap		Paired *t*-Test	Mean ± SE Moths per Trap		Paired *t*-Test
				1X PH	CM4K		1X PH	CM4K	
9a	Apple	Yes	26	12.5 ± 2.7 A	13.1 ± 2.2 A	*t*_25_ = 0.28, *p* = 0.7840	29.6 ± 4.9 A	31.5 ± 5.8 A	*t*_17_ = 0.23, *p* = 0.8177
	Apple	No	24	3.7 ± 1.5 B	29.0 ± 13.4 A	*t*_23_ = 2.10, *p* = 0.0469	11.1 ± 3.9 B	35.3 ± 7.6 A	*t*_23_= 3.78, *p* = 0.0010
9b	Pears	Yes	16	18.8 ± 4.1 A	17.3 ± 3.8 A	*t*_15_ = −0.46, *p* = 0.6544	34.4 ± 6.8 A	25.3 ± 6.1 A	*t*_15_ = −1.02, *p* = 0.3221
	Pears	No	11	5.5 ± 1.8 B	14.7 ± 3.7 A	t_10_ = 3.50, *p* = 0.0057	5.0 ± 1.9 A	14.3 ± 6.7 A	*t*_10_ = 1.73, *p* = 0.1134
10a	Apple	Yes	12	-	-	-	2.3 ± 0.5 B	8.5 ± 1.7 A	*t*_11_= 4.54, *p* = 0.0008
	Apple	No	41	10.0 ± 1.8 B	50.8 ± 7.3 A	*t*_40_ = 5.89, *p* < 0.0001	20.4 ± 3.6 B	55.0 ± 9.2 A	*t*_40_ = 5.26, *p* < 0.0001
10b	Pears	Yes	16	-	-	-	5.8 ± 1.0 B	10.8 ± 1.7 A	*t*_15_ = 2.73, *p* = 0.0154
	Pears	No	16	10.1 ± 1.8 B	33.7 ± 6.5 A	*t*_15_ = 4.01, *p* = 0.0011	12.6 ± 3.0 B	37.8 ± 5.8 A	*t*_15_ = 4.92, *p* = 0.0002

Row means followed by different uppercase letters were significantly different, paired-sample *t*-test, *p* < 0.05. All trials were conducted in organic and conventional apple and pear orchards.

## Data Availability

Data supporting the results are openly available in the public repository Zenodo at https://doi.org/10.5281/zenodo.18794034 (accessed on 26 February 2026).

## Supplementary Materials

The following supporting information can be downloaded at: https://www.mdpi.com/article/10.3390/insects17040354/s1, Figure S1: Photos of the various orange delta and a white round traps used in field trials of codling moth monitoring; Figure S2: Visual comparison of organic apple orchards with weed management (A) versus minimal weed management (B); Figure S3: Example of CM4K-baited delta trap liner’s non-target catch of *D. brevis* in an organic pear orchard, 2025; Figure S4: Photo of a typical sticky liner from a delta trap baited with CM4K lure showing the high selectivity for codling moth and few non-targets.
