# Supplementary figures and images for: Optimizing UV-A Solar-Powered Lights to Enhance Lures for Codling Moth, Cydia pomonella L. (Lepidoptera: Tortricidae)
Source: Insects. 2026 Mar 24;17(4):354. doi: 10.3390/insects17040354 (PMC13116518; doi:10.3390/insects17040354)

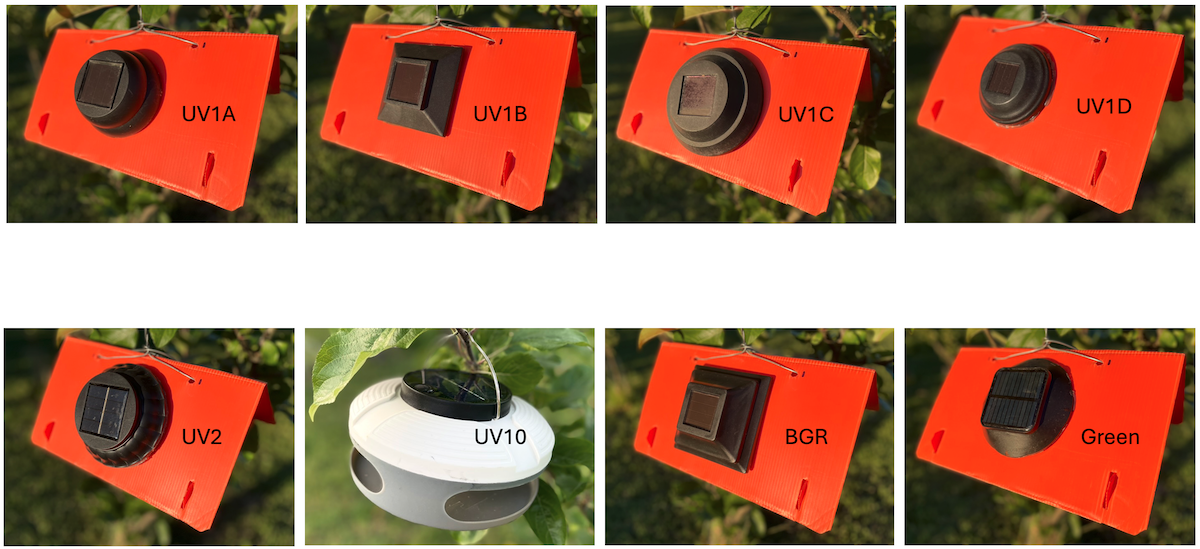

Supplement: Supplementary file 1 [file insects-17-00354-s001.zip › Figure S1.png]

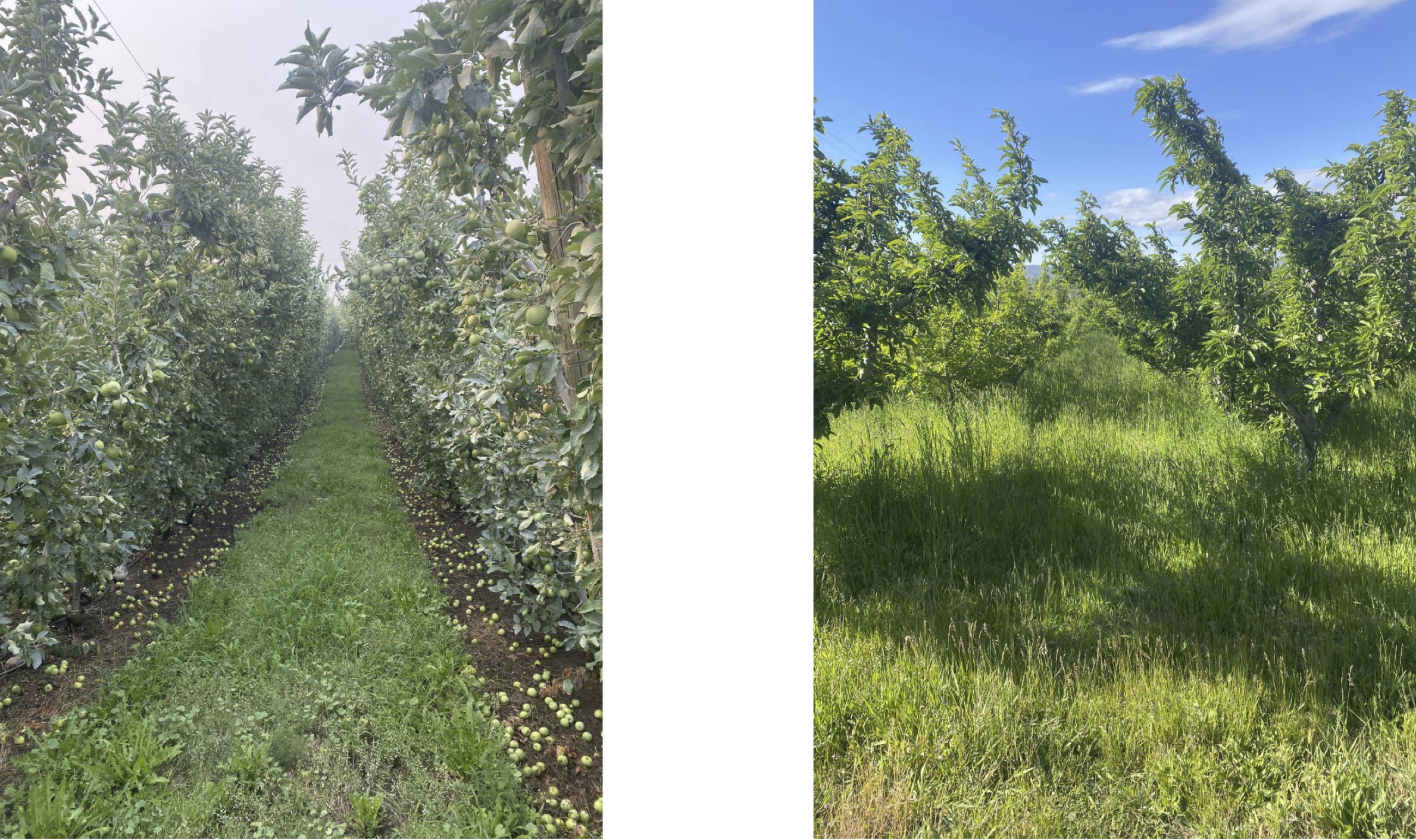

Supplement: Supplementary file 1 [file insects-17-00354-s001.zip › Figure S2.jpg]

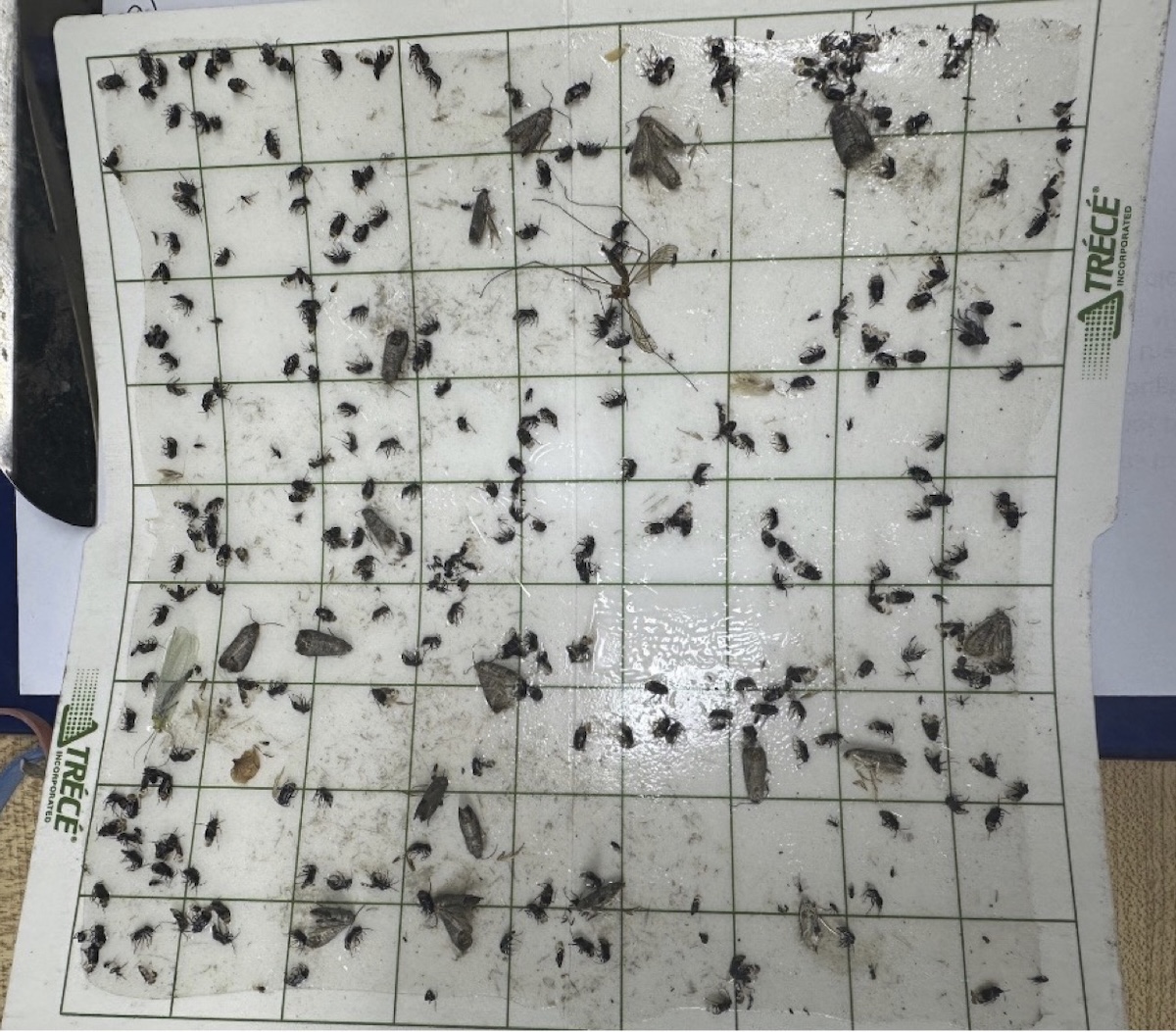

Supplement: Supplementary file 1 [file insects-17-00354-s001.zip › Figure S3.jpg]

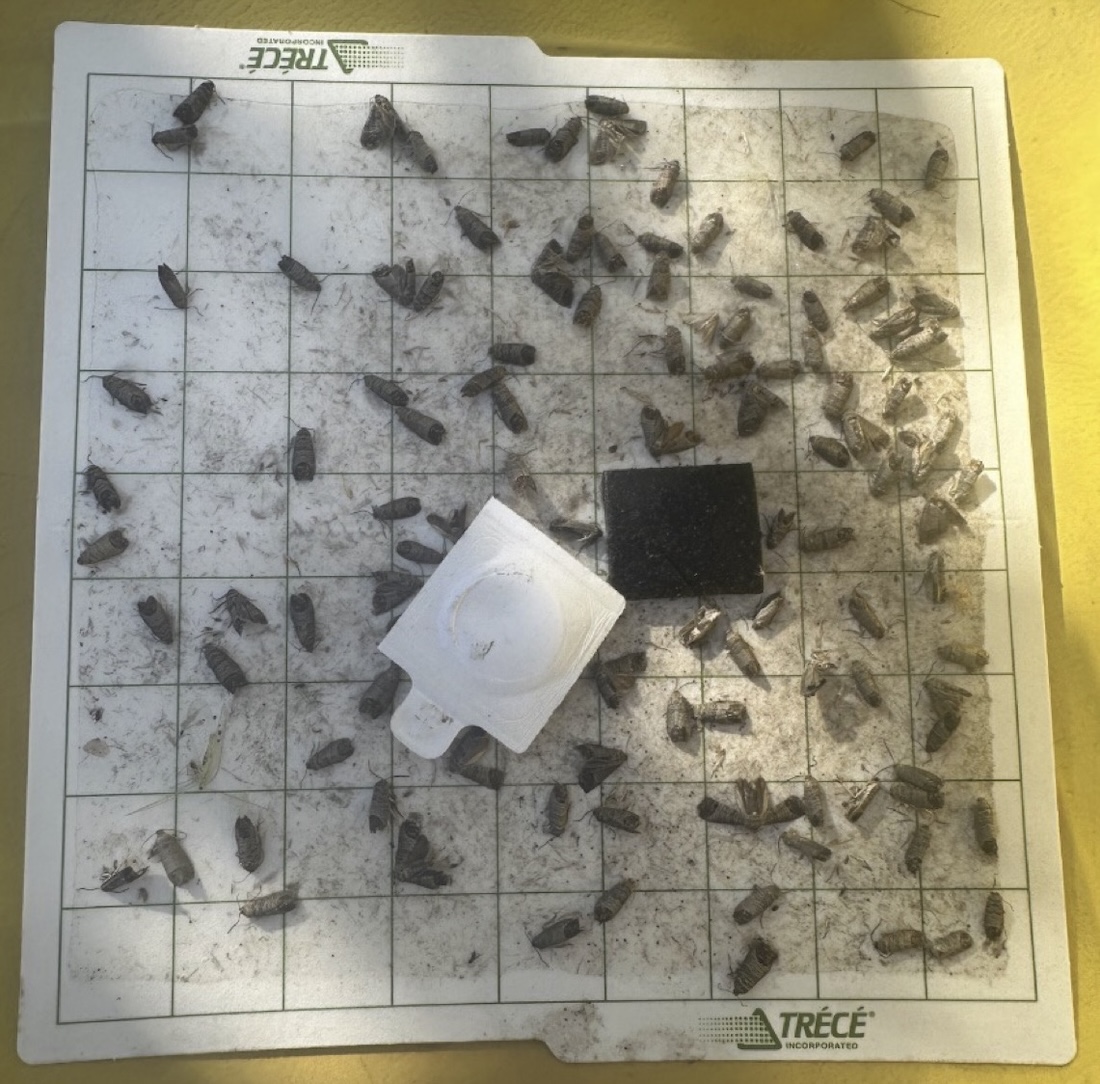

Supplement: Supplementary file 1 [file insects-17-00354-s001.zip › Figure S4.jpg]
